# Robotic-assisted cholecystectomy in children is associated with faster gastrointestinal recovery: a comparative study

**DOI:** 10.3389/fped.2026.1880433

**Published:** 2026-07-06

**Authors:** M. Di Mitri, M. M. Cantagalli, A. Morabito, A. Brucculeri, J. Belli1, S. Muscolino, R. Lo Piccolo, M. Di Maurizio, E. Ciardini, R. Coletta

**Affiliations:** 1 Department of Pediatric Surgery, Meyer Children’s Hospital, Florence, Italy; 2 School of Pediatric Surgery, University of Florence, Florence, Italy; 3 Department of Neurosciences, Psychology, Drug Research and Child Health (NEUROFARBA), University of Florence, Florence, Italy; 4 Radiology Unit, Meyer Children’s Hospital IRCCS, Florence, Italy

**Keywords:** cholecystectomy, pediatric cholecystectomy, pediatric hepatobiliary surgery, pediatric robotic surgery, robotic-assisted surgery

## Abstract

**Background:**

The role of robotic-assisted cholecystectomy in paediatric patients remains incompletely defined, particularly regarding postoperative recovery and gastrointestinal function. This study aimed to compare perioperative outcomes and early recovery between robotic-assisted and conventional laparoscopic cholecystectomy in children.

**Methods:**

A single-centre retrospective comparative cohort study was conducted at a tertiary paediatric referral centre. Consecutive patients (≤18 years) undergoing cholecystectomy for benign gallbladder disease were included and divided into two groups based on surgical approach: pre-robotic (January 2023–December 2024) and robotic (January 2025–December 2025). Demographic, intraoperative, and postoperative outcomes were analysed. Continuous variables were compared using the Mann–Whitney U test and categorical variables using Fisher’s exact test.

**Results:**

A total of 28 patients were included (13 pre-robotic, 15 robotic). Baseline characteristics were comparable between groups. Median operative time was significantly longer in the robotic group (2:22 h vs. 1:33 h; *p* < 0.001), while length of hospital stay was significantly shorter (2 vs. 4 days; *p* = 0.003). No conversions to open surgery or surgical site infections were observed. Postoperative complications were rare and comparable between groups (7.7% vs. 0%; *p* = 0.46). Analgesic requirements, VAS scores, and PEWS values were similar. Early gastrointestinal recovery was significantly improved in the robotic group. Early oral feeding within 24 h occurred in 100% of robotic cases versus 69.2% in the pre-robotic group (*p* = 0.041). Passage of flatus within 24 h was observed in 93.3% versus 30.8%, respectively (*p* = 0.002).

**Conclusions:**

Robotic-assisted cholecystectomy is a safe and feasible approach in paediatric patients, with perioperative outcomes comparable to conventional laparoscopy. Despite longer operative times, the robotic approach was associated with shorter hospital stay and faster recovery of bowel function. These findings suggest a potential advantage of robotic surgery in promoting early postoperative recovery. Larger prospective studies are needed to confirm these results and define the role of robotics in paediatric cholecystectomy.

## Introduction

Gallbladder disease in children includes a heterogeneous spectrum of conditions, most commonly cholelithiasis, biliary sludge, gallbladder dyskinesia, cholecystitis, and, less frequently, gallbladder polyps or congenital biliary abnormalities. In paediatric patients, gallstone formation may be associated with haemolytic disorders, obesity, rapid weight loss, parenteral nutrition, prematurity, cystic fibrosis, ileal disease or resection, and specific pharmacological exposures. The underlying pathophysiology varies according to the aetiology, but generally involves alterations in bile composition, gallbladder motility, cholesterol supersaturation, pigment stone formation, or impaired enterohepatic circulation (1). Initial management depends on symptoms, disease severity, and underlying aetiology. Conservative treatment may include observation in asymptomatic patients, analgesia, antibiotics in selected cases of acute inflammation, dietary counselling, and treatment of associated metabolic or haemolytic conditions. However, cholecystectomy remains the definitive treatment for symptomatic gallbladder disease, recurrent biliary pain, complications such as cholecystitis or pancreatitis, and selected structural lesions such as gallbladder polyps. Consequently, cholecystectomy is now among the most common hepatobiliary procedures in paediatric surgery (2).

Laparoscopic cholecystectomy is still the standard of care for gallbladder disease (3). Since its introduction in the early 1990s, minimally invasive surgery has largely replaced the open approach in both adult and paediatric populations, becoming one of the earliest procedures in which laparoscopy supplanted open surgery. Its advantages include reduced postoperative pain, shorter hospital stay, faster recovery, and improved cosmetic outcomes (4, 5).

More recently, robotic surgery has emerged as a further evolution of minimally invasive techniques (6). Robotic platforms offer several potential advantages over conventional laparoscopy, including three-dimensional high-definition visualization, tremor filtration, improved ergonomics, and wristed instruments that allow greater freedom of movement (7). These technical features may facilitate precise dissection and improve surgical dexterity, particularly in complex procedures or in anatomically challenging patients (8, 9).

The rationale for comparing robotic-assisted and conventional laparoscopic cholecystectomy in children lies in the need to determine whether the technical advantages of robotic surgery translate into measurable clinical benefits in a procedure for which laparoscopy already represents the standard of care. Robotic platforms provide three-dimensional visualisation, improved ergonomics, tremor filtration, and wristed instruments, which may facilitate precise dissection around Calot's triangle. Conversely, the robotic approach is associated with longer setup and docking times, higher costs, and a learning curve, making its role in routine paediatric cholecystectomy still uncertain.

While robotic surgery has gained widespread acceptance in adult hepatobiliary procedures, its role in paediatric surgery remains less clearly defined (10). Concerns related to operative time, costs, and the limited availability of robotic platforms have contributed to slower adoption in children.

Despite growing interest, data comparing robotic-assisted and conventional laparoscopic cholecystectomy in paediatric patients remain limited. In particular, evidence regarding postoperative recovery, pain control, and early return of bowel function is still evolving (11).

## Materials and methods

### Study design

This single-centre retrospective comparative cohort study was conducted at Meyer Children’s Hospital IRCCS, a tertiary paediatric referral centre in Florence, Italy, to evaluate the clinical impact of introducing robotic-assisted surgery for cholecystectomy in paediatric patients.

Patients were grouped into two consecutive cohorts based on surgical approach: a pre-robotic period (January 2023–December 2024) and a robotic period (January 2025–December 2025).

The study was approved by the local ethics committee and conducted in accordance with institutional standards for retrospective clinical research. [ROB-PED Study. *Robotic Pediatric Trial (ROB-PED)*. ClinicalTrials.gov Identifier: NCT07438704. Bethesda (MD): National Library of Medicine (US). Available from: https://clinicaltrials.gov/study/NCT07438704?term=NCT07438704&rank=1].

### Patient selection

Patients were identified through a retrospective review of the institutional surgical database and electronic medical records.

Consecutive patients aged ≤18 years who underwent cholecystectomy for benign gallbladder disease were identified through a retrospective review of the institutional surgical database and electronic medical records and were included to minimise selection bias.

Patients were excluded in case of incomplete perioperative data, open or emergency procedures.

A minimum follow-up of 30 days was required to evaluate early postoperative outcomes, including complications and reinterventions.

### Surgical technique

Robotic-assisted cholecystectomy was performed using a DaVinci XI robotic surgical platform according to the manufacturer's recommendations, with port placement adapted to patient size and body habitus. Patients were placed in the supine position under general anaesthesia. After creation of pneumoperitoneum, the patient was positioned in reverse Trendelenburg with slight left tilt to optimise exposure of the right upper quadrant.

A multi-port configuration was used. Trocar placement included three 8-mm robotic ports and one 12-mm assistant/extraction port. Ports were positioned under direct vision and spaced as widely as permitted by the patient's abdominal dimensions to minimise external arm collision and ensure adequate triangulation toward the hepatocystic triangle. The camera port was positioned to provide a central view of the operative field, while the working robotic ports were placed to allow bimanual dissection and gallbladder retraction. The assistant port was used for suction, clip introduction when required, and specimen retrieval.

**Figure 1:**
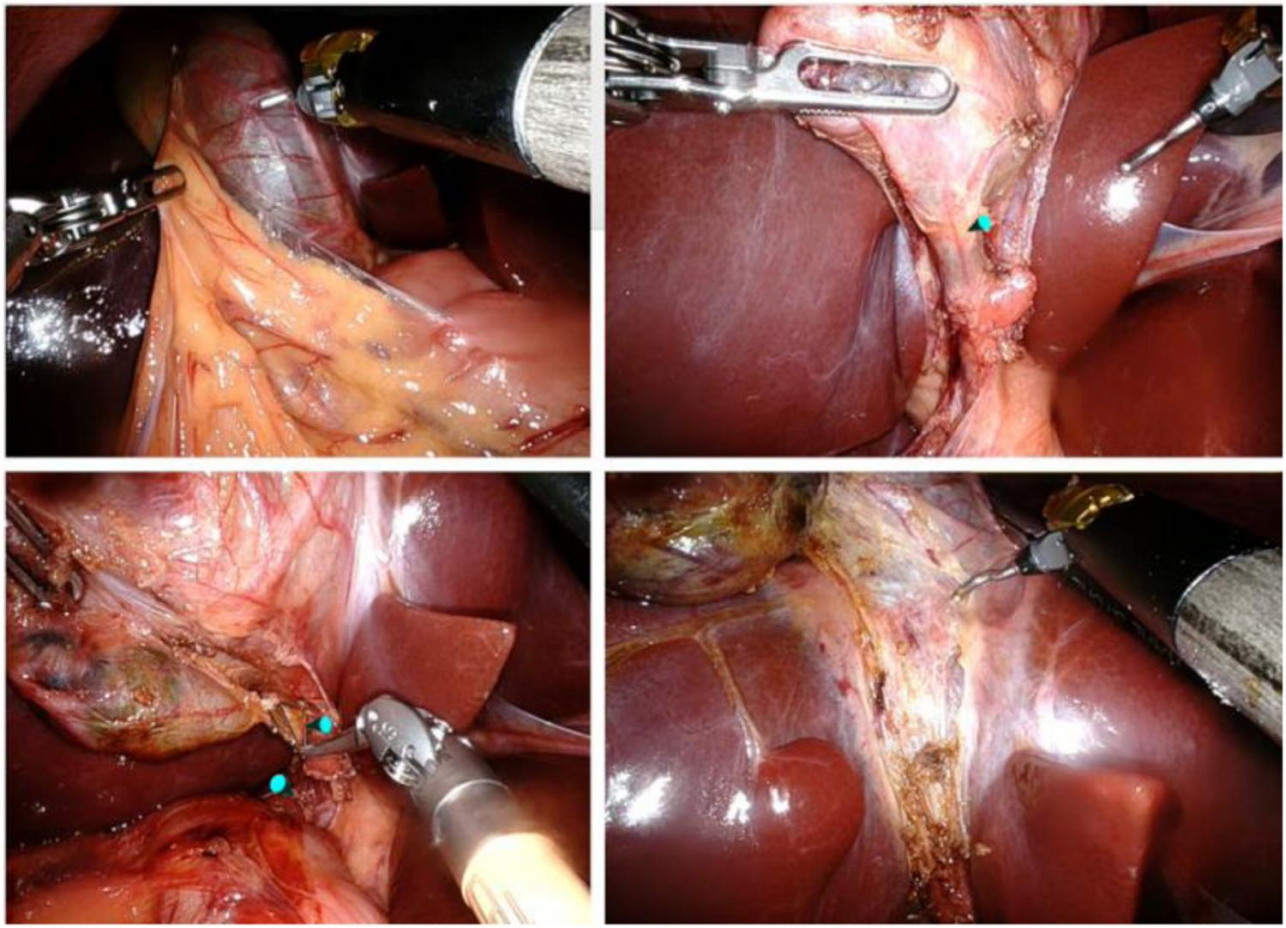
Intraoperative images of robotic cholecystectomy: exposure of Calot's triangle.

After docking of the robotic cart, the gallbladder fundus was retracted cranially and laterally to expose Calot's triangle. Dissection was performed robotically until the critical view of safety was obtained. The cystic duct and cystic artery were then clipped and divided. The gallbladder was dissected from the liver bed using electrocautery, haemostasis was checked, and the specimen was retrieved through the assistant/extraction port. Port sites were closed according to standard institutional practice. Representative intraoperative findings are shown in Figure 1.

### Data collection

Demographic, anthropometric, intraoperative, and postoperative data were extracted from electronic medical records. Collected demographic and anthropometric variables included age, weight, height, and body mass index (BMI). Surgical variables comprised conversion to open surgery and operative time. Postoperative outcomes included length of hospital stay (LOS), surgical site infection (SSI), postoperative complications, reoperation within 30 days, and postoperative analgesic consumption. Analgesic use was recorded as the number of administered doses of paracetamol and non-steroidal anti-inflammatory drugs, as well as the requirement for opioid rescue therapy.

Complications were defined as any adverse event occurring within 30 days after surgery. Reoperation was defined as a return to the operating room within 30 days of the index procedure. Pain intensity was assessed using the Visual Analogue Scale (VAS) and recorded as the maximum daily value during the first three postoperative days. Clinical stability was evaluated using the Paediatric Early Warning Score (PEWS), with maximum daily values recorded over the same postoperative interval.

Perioperative and postoperative management protocols, including analgesic regimens, feeding strategies, and discharge criteria, were standardised and remained consistent throughout the study period.

### Statistical analysis

For the purpose of this study, benign gallbladder disease was defined as non-malignant gallbladder pathology requiring elective cholecystectomy. Included conditions were symptomatic cholelithiasis and gallbladder polyp. Patients undergoing emergency procedures, open surgery, or cholecystectomy for malignant or suspected malignant disease were excluded.

No formal *a priori* sample size calculation was performed because of the retrospective design, the limited annual volume of paediatric cholecystectomy, and the early implementation phase of the robotic programme. Instead, the study included all consecutive eligible patients treated during the predefined study periods. Therefore, the analysis should be interpreted as an exploratory single-centre implementation study aimed at describing feasibility, safety, and early postoperative outcomes rather than as a powered confirmatory comparison.

Continuous variables were reported as median and interquartile range because of the small sample size and the expected non-normal distribution of several perioperative variables, including operative time and length of stay. Accordingly, comparisons between groups were performed using the Mann–Whitney U test rather than the independent-samples t-test, as this non-parametric test does not require the assumption of normality. Categorical variables were compared using Fisher's exact test instead of the chi-square test because of the small sample size and low expected cell frequencies in several outcome categories.

## Results

A total of 28 cholecystectomies were included in the analysis, with 13 procedures performed during the pre-robotic period and 15 during the robotic period. When annualised, the surgical volume corresponded to approximately 6.5 eligible cases per year during the pre-robotic period and 15 eligible cases during the robotic period. This difference reflects the before–after nature of the study and may be related to changes in institutional referral patterns, case scheduling, and implementation of the robotic programme after its introduction. Baseline demographic and anthropometric characteristics were comparable between the two groups. Median age at surgery was 15 years (IQR 13–16) in the pre-robotic group and 14 years (IQR 11–15) in the robotic group (*p* = 0.29). Median body weight was 50.7 kg (IQR 43–61) and 57.0 kg (IQR 41–65) in the pre-robotic and robotic groups, respectively (*p* = 0.52).

Similarly, BMI values were comparable between groups, with a median of 19.9 kg/m^2^ (IQR 18.7–21.2) in the pre-robotic cohort and 20.7 kg/m^2^ (IQR 18.8–24.6) in the robotic cohort (*p* = 0.58) (Table 1).

**Table 1:** Baseline demographic and clinical characteristics.

Variable	Pre-robotic (*n* = 13)	Robotic (*n* = 15)	*p*-value
Age, years (median, IQR)	15 (13–16)	14 (11–15)	0.29
Weight, kg (median, IQR)	50.7 (43–61)	57.0 (41–65)	0.52
BMI, kg/m^2^ (median, IQR)	19.9 (18.7–21.2)	20.7 (18.8–24.6)	0.58
Indication: Cholelithiasis, *n* (%)	13 (100%)	14 (93.3%)	–
Indication: Gallbladder polyp, *n* (%)	0	1 (6.7%)	–

The main surgical indication was cholelithiasis, accounting for 27 cases (96.4%), while one case (3.6%) was performed for a gallbladder polyp. No conversions to open surgery were recorded in either group.

A significant difference was observed in the length of hospital stay (LOS). Median LOS decreased from 4 days (IQR 3–4) in the pre-robotic group to 2 days (IQR 2–3) in the robotic group (*p* = 0.003). Conversely, operative time was significantly longer in the robotic group, with a median duration of 2:22 h (IQR 2:10–2:33) compared with 1:33 h (IQR 1:04–1:52) in the pre-robotic group (*p* < 0.001). No surgical site infections were reported in either group. One postoperative complication occurred in the pre-robotic group (7.7%) and consisted of postoperative bleeding requiring return to the operating room for surgical haemostasis, whereas none were recorded in the robotic cohort (*p* = 0.46) (Table 2).

**Table 2:** Surgical outcomes.

Outcomes	Pre-robotic (*n* = 13)	Robotic (*n* = 15)	*p*-value
Operative time (h:min, median, IQR)	1:33 (1:04–1:52)	2:22 (2:10–2:33)	<0.001
Length of stay, days (median, IQR)	4 (3–4)	2 (2–3)	0.003
Conversion to open, *n* (%)	0 (0%)	0 (0%)	—
Postoperative complications, *n* (%)	1 (7.7%)	0 (0%)	0.46
Surgical site infection, *n* (%)	0 (0%)	0 (0%)	—

Postoperative analgesic requirements were comparable between groups. Median paracetamol administration was 4 doses (IQR 4–6) in the pre-robotic group and 5 doses (IQR 4–6) in the robotic group (*p* = 0.46). Median NSAID consumption was 2 doses (IQR 0–3) and 2 doses (IQR 0–4) in the pre-robotic and robotic groups, respectively (*p* = 0.74). Rescue analgesia was required in 3 patients (23.1%) in the pre-robotic group and 4 patients (26.7%) in the robotic group (*p* = 1.00).

Pain assessment using the VAS scale showed no significant differences during the first three postoperative days. Median VAS scores at postoperative day 1 were 2 (IQR 0–5) in the pre-robotic group and 4 (IQR 0–6) in the robotic group (*p* = 0.32). Values remained low and comparable on postoperative days 2 and 3. Similarly, PEWS scores, used to assess clinical stability, were comparable between groups across all postoperative days, indicating similar postoperative recovery profiles.

Recovery of bowel function was significantly faster in the robotic group. Early oral feeding within 24 h occurred in 9 of 13 patients (69.2%) in the pre-robotic group and in all patients in the robotic group (15/15; 100%) (*p* = 0.041). The absolute difference in early oral feeding was 30.8%, further supporting a consistent trend towards faster gastrointestinal recovery. Similarly, passage of flatus within 24 h was observed in 4 of 13 patients (30.8%) in the pre-robotic group compared with 14 of 15 patients (93.3%) in the robotic group (*p* = 0.002). This corresponded to an absolute difference of 62.5% in early flatus between groups, suggesting a clinically relevant effect despite the limited sample size (Table 3).

**Table 3:** Postoperative recovery and analgesia.

Outcome	Pre-robotic (*n* = 13)	Robotic (*n* = 15)	*p*-value
Paracetamol doses (median, IQR)	4 (4–6)	5 (4–6)	0.46
NSAID doses (median, IQR)	2 (0–3)	2 (0–4)	0.74
Rescue analgesia, *n* (%)	3 (23.1%)	4 (26.7%)	1.00
VAS POD1 (median, IQR)	2 (0–5)	4 (0–6)	0.32
Early oral feeding ≤24 h, *n* (%)	9 (69.2%)	15 (100%)	0.041
Early flatus ≤24 h, *n* (%)	4 (30.8%)	14 (93.3%)	0.002

## Discussion

A distinctive contribution of this study lies in the observation of earlier postoperative bowel recovery in the robotic group, with both earlier oral feeding and earlier passage of flatus within 24 h. This aspect has been largely overlooked in previous pediatric comparative studies, which have predominantly focused on operative time, complications, readmissions, and length of stay (11, 12).

Although early oral feeding and passage of flatus may be influenced by postoperative care protocols, both cohorts were managed within the same institutional setting, supporting the internal consistency of the comparison. These findings suggest that early gastrointestinal recovery may be a sensitive endpoint for detecting subtle differences between minimally invasive surgical approaches in children.

By explicitly examining early gastrointestinal recovery, our findings extend the current evidence base and highlight a potentially relevant dimension of postoperative recovery that has not been systematically assessed to date. Although this result should be interpreted with caution, it may suggest that the enhanced dexterity, three-dimensional visualisation, and more refined tissue handling associated with robotic surgery contribute to a faster physiological recovery. This interpretation remains hypothesis-generating and warrants confirmation in prospective studies adopting standardised enhanced recovery protocols (13, 14).

More broadly, our findings corroborate that robotic-assisted cholecystectomy represents a safe and feasible alternative to conventional laparoscopy in pediatric patients, with a perioperative profile that is overall comparable between the two approaches. In our series, the robotic group demonstrated a shorter length of stay alongside the previously noted faster recovery of bowel function, while operative time was significantly longer. This pattern is consistent with the current pediatric literature, which supports the feasibility of the robotic approach but does not yet demonstrate a clear and systematic advantage over laparoscopy in short-term surgical outcomes (11, 12).

The comparable safety profile observed in our cohort is fully aligned with existing evidence. Prior pediatric comparative studies have consistently reported no major differences in postoperative complications, readmissions, or overall clinical success between robotic and laparoscopic cholecystectomy (12, 15).

This convergence of findings supports the interpretation that robotic surgery is at least noninferior to laparoscopy from a safety perspective. In parallel, recent pooled analyses have reached similar conclusions, showing no significant differences in overall safety and effectiveness between the two techniques in children (11).

The shorter hospital stay observed in the robotic group is clinically relevant, although it should be interpreted cautiously. In our series, median postoperative stay decreased from 4 to 2 days following the introduction of the robotic platform. While some pediatric studies report similar lengths of stay across techniques, pooled evidence suggests at least a tendency toward earlier discharge with robotic procedures, even when statistical significance is not consistently achieved (11, 12).

It is also important to acknowledge that institutional factors, including evolving postoperative pathways, discharge practices, and increasing team experience, may have contributed to this finding.

By contrast, the longer operative time observed in the robotic cohort is entirely consistent with prior evidence and remains the most robust difference reported in the literature. Pediatric studies have repeatedly shown longer operative times for robotic cholecystectomy, largely driven by docking, setup, and the learning curve associated with platform adoption (12, 16).

This interpretation is further supported by the broader pediatric robotic literature, where operative time is recognized as a key marker of institutional experience (17).

Accordingly, the longer duration observed in our study likely reflects the early implementation phase rather than an intrinsic limitation of the robotic technique.

Pain-related outcomes were comparable between groups, with no significant differences in analgesic requirements, VAS scores, or PEWS. This finding mirrors existing evidence, as prior pediatric studies have generally reported similar postoperative pain profiles between robotic and laparoscopic cholecystectomy, without consistent reductions in analgesic use in the robotic group (12, 16).

The most recent meta-analysis confirms this pattern, indicating that while robotic assistance does not increase postoperative discomfort, convincing evidence of a clinically meaningful analgesic benefit is still lacking (11).

These findings should be interpreted within the broader context of pediatric cholecystectomy, where conventional laparoscopy remains the standard of care. Systematic reviews consistently demonstrate that laparoscopic cholecystectomy in children is safe, effective, and associated with excellent outcomes. In this setting, the potential value of robotic surgery does not lie in replacing an already well-established approach, but rather in identifying specific perioperative or recovery-related advantages that may justify its adoption in selected contexts.

## Limitations

This study has several limitations that should be acknowledged. First, the retrospective, single-centre design limits the generalisability of the findings. As the study was conducted in a tertiary paediatric referral centre in Florence, Italy, the results may reflect local referral patterns, institutional protocols, surgical expertise, and population characteristics, and should therefore not be directly extrapolated to other geographical or healthcare settings.

Second, the comparison was performed across two consecutive time periods, and the number of eligible cases differed between the two cohorts despite the shorter duration of the robotic period. This discrepancy may reflect organisational changes following the introduction of the robotic programme, including increased referral, case centralisation, or scheduling patterns. Therefore, the study should be interpreted as a before–after implementation analysis rather than a purely technique-driven comparison.

Third, the relatively small sample size reduces the statistical power to detect differences in infrequent outcomes, such as postoperative complications, and limits the precision of effect estimates. This is particularly relevant in the paediatric setting, where case volumes are inherently lower compared with adult series.

Fourth, the robotic cohort represents the early phase of institutional adoption of the platform. As such, operative times are likely influenced by the learning curve associated with docking, setup, and team coordination. Conversely, the observation of improved postoperative recovery despite this early implementation phase may suggest that the reported benefits are conservative estimates and could become more pronounced as experience increases.

Finally, no formal cost analysis was performed. This represents an important limitation, particularly because economic sustainability remains one of the main concerns regarding the implementation of robotic surgery in paediatric practice. In the present study, a cost evaluation was not included because of the small sample size, the retrospective design, and the fact that the robotic cohort represents the initial phase of institutional adoption of the platform. During this learning-curve period, operative time, docking time, instrument use, and team organisation may not yet reflect a stable or mature robotic programme, making any cost comparison potentially unreliable. Future prospective studies with larger cohorts should include structured economic analyses, integrating direct procedural costs, operating room time, length of hospital stay, postoperative recovery, and resource utilisation, in order to better define the overall clinical and economic impact of robotic-assisted cholecystectomy in children (12).

## Conclusions

Our findings suggest a potential recovery-related benefit of robotic-assisted cholecystectomy in children, namely an earlier recovery of bowel function, with faster oral feeding and earlier passage of flatus compared with laparoscopy. This aspect, rarely addressed in prior pediatric studies, represents the most distinctive contribution of our analysis and suggests a potential benefit of the robotic approach in promoting faster physiological recovery after surgery.

Overall, our experience confirms that robotic-assisted cholecystectomy is a safe and feasible option in the paediatric population. While the robotic approach was associated with longer operative times, it also showed a shorter hospital stay and, notably, improved early postoperative recovery, with comparable pain control and complication rates between techniques.

Further large-scale, multicentre prospective studies are warranted to better define the role of robotics in this setting, particularly to validate its impact on recovery outcomes, assess cost effectiveness, and account for the influence of the learning curve.

## Data Availability

The datasets presented in this study can be found in online repositories. The names of the repository/repositories and accession number(s) can be found in the article/Supplementary Material.
